# Ergot; Its Natural History and Uses as a Therapeutic Agent

**Published:** 1861-03

**Authors:** 


### ERGOT; ITS NATURAL HISTORY AND USES AS A THERAPEUTIC AGENT.

PROF. E. N. CHAPMAN, of the Long Island College Hospital, concludes an excellent article in the *Medical and Surgical Reporter* (Jan. 19th) with the following summary:

1st. The active principles of ergot are the products of a plant, the oidium abortifaciens. These are peculiar to it, and are in no wise more dependent on the grain-germ, than any plant is on the soil that affords it nourishment.

2nd. These principles—not as yet isolated—are, probably, two in number; one of which, contained in the oil, produces narcotic symptoms, and eventually gangrene; the other, remaining in the residue after the extraction of the oil, acts by stimulation on the motor tract of the spinal marrow at that limited portion whence the uterus and contiguous muscles receive their nerves of motion.

3d. The oil is alone efficacious in any form of hemorrhage, excepting in that from enlarged uterus, and we can only look for favorable results when the system has been brought under its influence, as shown by a narcotic impression on the brain and heart.

4th. So hazardous a treatment cannot be countenanced; since we possess other remedies of known virtues, not only more reliable, but unattended with any risk.

5th. The watery preparations, which, lacking the oil, can be given many days in succession, will act on the motor tract of the spinal marrow, but will not induce ergotism or other evidence of a narcotic poison.

6th. Ergot of good quality causes the uterus to contract with great certainty whenever its cavity is distended, either by a fœtus or a morbid growth. These contractions are tonic and continuous, with periods of remissions and exacerbations.

11th. Hence, this remedy may originate uterine pains at any period of gestation, or cause the expulsion of clots, hydatyds, or any other unattached substance.

12th. Likewise, after labor, it will check an excessive flow by closing the mouths of the bleeding vessels through a condensation of the muscular fibres of the uterus. In relaxed conditions it will be, on the same principle, beneficial in after-pains attended with a copious show.

13th. Since we know of no other mode by which ergot can immediately arrest hemorrhage from the pregnant uterus, it should be avoided in all cases where a hope still remains of saving the conception, and its agency invoked only when the fœtus is dead or the safety of the mother becomes our first duty.

14th. In premature labor, induced by ergot, or in labor at the full period, where this drug is given, the child's life is jeopardized in all cases, where for a considerable period previous to the delivery, the uterus is thrown into violent spasmodic action; since thus the supply of oxygen will be most effectually cut off.

15th. Where the child is viable, the ergot should never be used as an expulsive agent to increase pains, already powerful, or overcome resistance from the soft parts of the mother.

16th. It may be required in cases of inertia of the uterus where, with all the parts relaxed, we are quite certain, both from the present appearances and the history of former labors, that the child would be born in four or five vigorous natural pains.

17th. The dangers to the mother from the use of ergot, arise from the uterus thus stimulated, rupturing the unyielding parts in its transit. Thus the neck of the uterus, the bladder or the perineum may receive serious injury.

18th. Rupture of the uterus could hardly occur, excepting in a diseased organ.

19th. A premature fœtus from its small size and the yielding nature of the cranial bones could scarcely, under any uterine effort, cause damage to the soft parts of the mother.

